# Targeting tumor cells with antibodies enhances anti-tumor immunity

**DOI:** 10.1007/s41048-018-0070-2

**Published:** 2018-10-29

**Authors:** Zhichen Sun, Yang-Xin Fu, Hua Peng

**Affiliations:** 1 Key Laboratory of Infection and Immunity, Institute of Biophysics, Chinese Academy of Sciences, Beijing, 100101 China; 2 University of Chinese Academy of Sciences, Beijing, 100049 China; 3 Department of Pathology, University of Texas Southwestern Medical Center, Dallas, TX 75390 USA

**Keywords:** Tumor antigen, Targeting antibody, Innate immunity, Adaptive immunity, Cytokine, Tumor microenvironment

## Abstract

Tumor-targeting antibodies were initially defined as a group of therapeutic monoclonal antibodies (mAb) that recognize tumor-specific membrane proteins, block cell signaling, and induce tumor-killing through Fc-driven innate immune responses. However, in the past decade, ample evidence has shown that tumor-targeting mAb (TTmAb) eradicates tumor cells via activation of cytotoxic T cells (CTLs). In this review, we specifically focus on how TTmAbs induce adaptive anti-tumor immunity and its potential in combination therapy with immune cytokines, checkpoint blockade, radiation, and enzyme-targeted small molecule drugs. Exploring the mechanisms of these preclinical studies and retrospective clinical data will significantly benefit the development of highly efficient and specific TTmAb-oriented anti-tumor remedies.

## Introduction of Classical Tumor-Targeting Antibodies

The first generation of tumor-targeting antibodies approved by US Food and Drug Administration (FDA), including trastuzumab, cetuximab, and rituximab, were initially known as signal blockers to target oncogenic receptors of tumor cells and have great potential for effective cancer immunotherapy (Hynes and Lane 2005; Li *et al.* 2005). Later, Fc receptor (FcR) in immune cells was found to play an essential role in Ab-dependent cell cytotoxicity to tumor cells *in vivo* (Clynes *et al.* 2000; Musolino *et al.* 2008) and Complement-dependent cytotoxicity (CDC) (Teeling *et al.* 2004; van Meerten *et al.* 2006). Recently, we and others have reported the function of adaptive immunity in the Ab-mediated tumor regression (Abes *et al.* 2010; Mortenson *et al.* 2013; Park *et al.* 2010; Ren *et al.* 2017; Stagg *et al.* 2011; Yang *et al.* 2013b, 2014). With further understanding of tumor microenvironment (TEM), tumor-targeting Ab has been used to construct bispecific Ab and Ab–cytokine. In combination with checkpoint blockade Abs, radiation therapy, and traditional small molecule chemo- and targeted drugs, TTmAbs can be widely applied to break the immune tolerance and acquired resistance to long-term anti-tumor treatments for the final tumor elimination.

## Roles for Tumor-Targeting AB to Bridge Innate Immunity to CTL

### Anti-HER2 antibody

The human epidermal growth factor receptor 2 (HER2) is the homologue of the rat neu oncogene (HER2/neu). Along with HER1 (EGFR), HER3 and HER4, these proteins belong to the type I growth receptor family (Rajkumar and Gullick 1994). These transmembrane receptors, with molecular weights of approximately 170–185 kDa, are receptor tyrosine kinases that undergo homo- or hetero-dimerization when activated by related ligands (Olayioye *et al.* 2000; Rajkumar and Gullick 1994; Shepard *et al.* 2008), but the specific ligand for HER2/neu (ErbB2) is still not identified (Yarden and Pines 2012). This receptor signaling plays essential roles in multiple normal cellular processes of cell proliferation, differentiation, adhesion, motility, and apoptosis (Menard *et al.* 2003; Quaglino *et al.* 2008). However, HER2 has been found to be overexpressed in a quarter of breast cancers and is connected to higher malignancy, relapse rates, and mortality (Hudis 2007; Kiessling *et al.* 2002; Meric-Bernstam and Hung 2006; Slamon *et al.* 1987). In 1998, the FDA approved trastuzumab (Herceptin) for clinical trial in treating human breast cancer patients. Trastuzumab is a humanized monoclonal antibody containing the complementary regions of the murine antibody (clone 4D5) and Fc region of human IgG1 (Carter *et al.* 1992; Nahta and Esteva 2006). It binds to HER2 on the cell surface and has been proven to be an effective treatment for HER2/neu-positive breast cancers in multiple animal studies and clinical trials (Abramson and Arteaga 2011; Hudis 2007; Moasser 2007).

*In vitro* mechanistic studies have shown that the anti-HER2/neu antibody inhibits HER2/neu+ tumor growth, mainly via inducing G1 cell cycle arrest (Le *et al.* 2005; Mittendorf *et al.* 2010). Antibody-dependent cellular cytotoxicity (ADCC) is also required for the antitumor effects of anti-HER2 therapy. NK cells induce ADCC after the anti-HER2/neu antibody engagement, resulting in HMGB-1 release and MyD88-dependent TLR stimulation (Clynes *et al.* 2000; Musolino *et al.* 2008). Park *et al.* firstly revealed that the adaptive immune system plays critical roles in anti-HER2/neu-mediated anti-tumor therapeutic effect (Park *et al.* 2010). They demonstrate that (1) the therapeutic effect of HER2/neu antibody is tumor-specific T-cell dependent; (2) effective anti-HER2/neu treatment achieves immune memory that resists the subsequent high dose tumor rechallenge; (3) anti-HER2/neu treatment results in enhanced CD8+ cell infiltration in the tumor tissue of mouse models and clinical patients.

### Anti-EGFR antibody

Epidermal growth factor receptor (EGFR) is another member of the type I growth receptor family. Although HER2 is characterized by lacking an identified ligand, EGFR has been found to have several other ligands besides EGF, such as transforming growth factor alpha (TGF-α) (Citri and Yarden 2006; Yarden and Sliwkowski 2001). The first FDA-approved anti-EGFR monoclonal antibodies, including cetuximab (Erbitux, a human–murine chimeric. Ennis *et al.* 1991) and panitumumab (a human monoclonal antibody), have been successfully used for the treatment of EGFR-expressing cancers (Yang *et al.* 2001). These monoclonal antibodies bind to the extracellular domain of EGFR, inhibiting receptor dimerization and downstream signaling (Burgess *et al.* 2003), and inducing receptor internalization, ubiquitinization, and degradation (Sunada *et al.* 1986). ADCC and CDC are also the tumor-killing mechanisms resulting from the binding of the monoclonal antibody (Kimura *et al.* 2007). Primary and acquired resistance becomes increasingly challenging for targeted therapy (Bardelli and Siena 2010; Cobleigh *et al.* 1999). Ab resistance mediated by mutations within targeted oncogenes or in genes related to oncogenic pathways has been broadly investigated (Misale *et al.* 2012; Yonesaka *et al.* 2011). These studies will provide important information for developing drugs that target the increasing intrinsic resistance in tumor cells after antioncogenic Ab treatment (Bostrom *et al.* 2009; Fayad *et al.* 2013; Hurvitz *et al.* 2013; Krop *et al.* 2012; Yoon *et al.* 2011). Nevertheless, Yang *et al.* focus on tumor-extrinsic resistance and propose a tumor-extrinsic strategy to bypass intrinsic Ab resistance by reactivating both innate and adaptive immune cells inside the tumor. Using Ab-sensitive TUBO (HER2/neu+) and Ab-resistant EGFR-transduced B16 mouse tumor models (Rovero *et al.* 2000), they found increased production of type I IFNs in the Ab-sensitive tumor model, in contrast to the Ab-resistant tumor model. These data suggest that enhanced type I IFN production was caused by Ab-induced oncogenic receptor and stress. Yang *et al.* are the first to point out that Type I IFNs are the cytokines essential for Ab-mediated tumor regression and tumor-targeting delivery of type I IFNs may induce stronger anti-tumor immune responses to overcome antibody resistance or tumor immune tolerance.

### Anti-CD20 antibody

Rituximab is a murine–human chimeric antibody that recognizes the human B-cell CD20 antigen featuring primary response rates up to 70% (Maloney *et al.* 1992, 1994, 1997a, b). Similar to cetuximab and panitumumab, rituximab can induce tumor-cell death via ADCC, CDC, and apoptosis of tumor cells *in vitro* and in animal models (Clynes *et al.* 2000; Maloney 2012). Rituximab was approved by the FDA in 1997 for treating non-Hodgkin B-cell lymphoma. Effective control of B-cell lymphoma by anti-CD20 in xenograft models indicates the importance of direct tumor killing or innate-mediated killing induced by this antibody. However, the function of the adaptive immune response was later discovered to also play essential roles in lymphoma clearance. Using the huCD20-EL4 tumor model, a murine T-cell lymphoma transfected with the human CD20 molecule, Abes and Xuan reported that by the CD4+, but not CD8+, T-cell immune responses may contribute to long-lasting protection by anti-human CD20 treatment (Abes *et al.* 2010; Xuan *et al.* 2010). Conversely, Ren *et al.* reported in 2016 that CD8+ T cells alone, but not CD4+ T cells, contributed to the effective anti-mouse CD20 Ab therapy in a syngeneic A20 B-cell lymphoma mouse model. In this study, they characterized how anti-CD20 treatment initiated a potent tumor-specific T-cell response for tumor control. Ab could kill some tumor cells through ADCC by macrophages that produce type I IFNs for cross-priming; IFN binds to interferon α/β receptor (IFNAR) and activates Dendritic Cells (DCs) to better process tumor antigens for cross-priming T cells in the DLN; tumor-specific CTLs travel back to the tumor site for tumor control. They further demonstrated the role of CTLA-4 in Tregs within advanced B-cell lymphoma in limiting anti-CD20-mediated tumor regression. Thus, anti-CTLA-4 and anti-CD20 combined treatment is a possible new clinical strategy in overcoming adaptive resistance and preventing relapse of B-cell lymphoma.

Overall, these studies reveal the essential contribution of adaptive immune responses in the early elimination and late resistance of TTmAb therapy. Most importantly, these studies have demonstrated that DCs are the major tolerized immune cells in tumors and DCs determine the immune-active or immunosuppressive status in TME. Targeting DCs will be another important strategy for improving the efficacy of cancer immunotherapy. This can be achieved by providing type I IFNs, the key players linking innate and adaptive antitumor immunity, to induce Ab-mediated tumor regression. Moreover, these studies raise the potential of using checkpoint blockades to overcome adaptive resistance in the future.

## Antibody Armed with Cytokines to Further Promote Adaptive Anti-tumor Immunity

Type I IFNs, including IFN-α, IFN-β, IFN-ε, IFN-κ, and IFN-ν, are a family of monomeric cytokines with multiple functions (Pestka *et al.* 2004). Type I IFNs are involved in regulating many aspects of innate and adaptive immune responses by affecting the activation, migration, differentiation, and survival of macrophages, NK cells, DCs, monocytes, and B/T cells. Type I IFNs have been demonstrated to improve the antigen cross-presentation ability of CD8a+ DCs (Diamond *et al.* 2011; Lorenzi *et al.* 2011).

Due to their direct anti-proliferation and pro-apoptosis effects, Type I IFNs have been used for melanoma and lymphoma treatments. Recently, our laboratory and other research groups have found that endogenous type I IFNs perform significant functions in various antitumor therapies (Deng *et al.* 2014; Sistigu *et al.* 2014; Woo *et al.* 2014).

However, administration of type I IFNs has not been efficient in clinical cancer therapy, because of its limited potency and severe side effects (Trinchieri 2010). It has been suggested that the delivery of exogenous type I IFN may dramatically impact both immune responses and tumor cell proliferation/survival (Tang *et al.* 2016; Trinchieri 2010).

Yang *et al.* proposed that type I IFNs play an essential and sufficient role to bridge innate and adaptive antitumor immune responses during Ab-based antitumor therapy (Yang *et al.* 2014). They developed Ab–IFNβ as an advanced therapeutic strategy for those Ab-resistant tumors. Their study demonstrates that Ab-resistant tumors can be efficiently controlled by employing Ab–IFNβ fusion proteins. IFNAR on DCs, but not on tumor cells or T cells was required for the targeting effect of Ab–IFNβ which increases DC cross-presentation and antitumor CTL function.

Another study demonstrated that Anti-CD20–IFNα fusion protein was more effective than anti-CD20 Ab alone for direct killing of type IFNα receptor (IFNAR)-positive lymphoma (Xuan *et al.* 2010). However, the function of IFNAR on host immune cells was not addressed. Using a syngeneic immunocompetent mouse model, Liao *et al.* observed that targeting lymphoma with IFNα abolished resistance of B-cell lymphoma to anti-CD20 Ab while also limiting interferon (IFN)-associated systemic toxicity in the host (Liao *et al.* 2017). Anti-CD20–IFNα fusion protein-mediated tumor control is dependent on existing tumor-infiltrating CD8+ T cells. Although resistant to direct killing induced by IFNα, IFN-exposed A20 lymphoma cells become the dominant APCs for the reactivation of CTLs in the tumor. Anti-CD20–IFNα also abolishes checkpoint blockade resistance in advanced B-cell lymphoma. Thus, this study indicates that anti-CD20–IFNα eradicates B-cell lymphoma by employing tumor cells as APCs to reactivate tumor-infiltrating CD8+ T cells and synergizing with anti-PD-L1 treatment.

LIGHT (“the homologous to lymphotoxin that exhibits inducible expression and competes with HSV glycoprotein D for binding to herpesvirus entry mediator, a receptor expressed on T lymphocytes”), also known as tumor necrosis factor superfamily member 14 (TNFSF14), is one of the co-stimulatory molecules for T-cell activation (Lee *et al.* 2006; Wang *et al.* 2009). LIGHT is predominantly expressed on the surface of immature dendritic cells (DCs) and activated T cells. Several studies have indicated that LIGHT signaling might increase lymphocyte infiltration in the tumor (Yu *et al.* 2004, 2007; Zou *et al.* 2012). Based on LIGHT activities, Tang *et al.* constructed an anti-EGFR–LIGHT (Ab–LIGHT) targeting into EGFR+ but anti-PD-L1-resistant tumor tissues (Tang *et al.* 2016). In the study, they demonstrated that LIGHT indeed induces lymphocyte infiltration and antitumor immunity in both mouse and human tumor model. They also proved that additional LIGHT treatment can promote the efficacy of checkpoint blockade therapies by enhancing lymphocyte infiltration. These data suggest that LIGHT could be used to increase the responsiveness to checkpoint blockades and other immunotherapies in non-T-cell-inflamed tumors.

Interleukin-2 (IL-2) is a pleiotropic cytokine that promotes proliferation of NK and T cells induced by antigen stimulation (Liao *et al.* 2013; Morgan *et al.* 1976). IL-2 was one of the first FDA-approved immunotherapy drugs for metastatic melanoma and renal cell cancer (Rosenberg 2014; Rosenberg *et al.* 1998). However, IL-2 immunotherapy has not been widely employed because of its short half-life *in vivo* and severe toxicity at a therapeutic dose (Chavez *et al.* 2009; Panelli *et al.* 2004; Skrombolas and Frelinger 2014). In addition, IL-2 induces proliferation of regulatory T cells (Tregs) through binding to IL-2R alpha that is preferentially expressed on Tregs (Ahmadzadeh and Rosenberg 2006; Jensen *et al.* 2009; Sim *et al.* 2014), which might be a major barrier for IL-2-mediated expansion of CTL. To limit systemic toxicity, antibody-based delivery of IL-2 (Ab-IL2) has been investigated (Becker *et al.* 1996; Du *et al.* 2013; Gillies *et al.* 2011; Gutbrodt *et al.* 2013, 2014; Yang *et al.* 2013a). Systemic delivery of IL-2 may activate T cells in lymphoid and non-lymphoid tissues. The anti-tumor function of IL-2 therapy is known to directly activate CTLs (Gutbrodt *et al.* 2014; Jackaman *et al.* 2003). However, IL-2 induced Treg inhibitory effects are also significant. Thus, there are tremendous research efforts in constructing the mutant IL-2 with tumor targeting that increases binding to CD8+ T cells and reduces affinity to Tregs, which can alter antitumor response in tumor microenvironment and activate tumor-specific CTLs, thus leading to significantly improved anti-tumor responses (De Luca *et al.* 2017; Hartimath *et al.* 2018; Zhu *et al.* 2015). Similarly, IL-21, IL-12, and IL-15 cytokines are under investigation for possibilities in achieving potent tumor-targeting effects with minor peripheral side effects.

In summary, these studies established new concepts for the Ab-based treatment, such as the Ab-IL2, Ab–IFNβ, and Ab–LIGHT fusion proteins, which stimulate or augment the tumor-specific CTL responses to deal with Ab resistance and relapse more effectively. Killing more tumor cells by enhanced CTLs can then create a positive feedback loop for anti-tumor immune responses. In addition, all these studies conclude that blocking inhibitory PD-L1 upregulated by Ab–cytokine treatment may further improve the antitumor effect via recruiting more Ab–cytokine molecules and open new avenues for future clinical cancer treatment.

## Antibodies Targeting to Immune Inhibitory Receptors and Combination Therapies

### Anti-CD47

CD47 is a major player of the ‘donot eat me’ signal (McCracken *et al.* 2015). The inhibitory phagocytic signaling is transduced when cell-surface CD47 binds signal-regulatory protein alpha (SIRPα) on phagocytic cells (Willingham *et al.* 2012). CD47 is overexpressed on tumor cells and considered as a marker for cancer prognosis (Chan *et al.* 2009; Jaiswal *et al.* 2009; Majeti *et al.* 2009). The characteristic overexpression of CD47 on tumor cells also makes it a potential target for antibody-driven immunotherapy. There are several ongoing clinical trials for anti-CD47 monoclonal antibodies that have yet to yield conclusions (Russ *et al.* 2018). Risk for toxicity and anti-tumor effectiveness from a CD47 blockade is still under observation. Tumor-associated macrophages were commonly reported to be the major anti-tumor phagocytes in xenograft transplantation models, where the role of adaptive immunity is excluded (Chao *et al* 2010, 2011; Willingham *et al.* 2012). However, using syngeneic immune complete cancer mouse models instead of transplanted xenografts, Liu *et al.* first demonstrated that the anti-tumor effects mediated by the CD47 blockade was mostly dependent on the tumor-specific CD8+ T cells. CD11c+ DCs, but not macrophages, are the major APCs for the cross-priming of CD8+ T cells in a STING signaling-dependent manner to further drive type I IFN production and CTL activation. This DC activation was not induced by the MyD88 Toll-like receptor signaling as previously reported. The discovery of adaptive antitumor immunity mediated by CD47-targeting Ab blockade sheds light on designing new strategies with anti-CD47 in conjunction with traditional chemotherapeutics and other targeted therapies.

### Ab in combination with checkpoint blockade

Cytotoxic T-lymphocyte-associated antigen-4 (CTLA4) and programmed death-1 (PD1) are two of the major co-inhibitory immune checkpoint molecules expressed on T cells. PD1 ligand (PDL1) is another immune inhibitory molecule expressed on dendritic cells, activated T cells, and tumor cells. Anti-CTLA4 and anti-PD1/PD-L1 monoclonal antibodies have been developed as checkpoint blockades to inhibit the suppressive function of CTLA4 or PD-1/PD-L1 (Pardoll 2012). Physiologically, these inhibitory molecules play important roles in protecting the host from autoimmune diseases (Keir *et al.* 2008; Nishimura *et al.* 2001). However, tumors take advantage of these pathways to escape antitumor immune responses (Dong *et al.* 2002; Iwai *et al.* 2002; Shin and Ribas, 2015). Nivolumab and pembrolizumab, two monoclonal antibodies targeting PD1, have shown impressive anti-tumor efficacy in melanoma and non-small cell lung cancer (NSCLC) (Brahmer *et al.* 2012; Robert *et al.* 2015a, b; Topalian *et al.* 2012). Ipilimumab, a first class anti-CTLA4 monoclonal antibody (Hodi *et al.* 2010; O’Day *et al.* 2007; Wolchok et al. 2010), is effective in about 10%–20% of patients (Maio *et al.* 2015; Schadendorf *et al.* 2015).

Studies have shown that checkpoint blockades can reverse T-cell suppression and improve the therapeutic effects. However, only a small number of patients are sensitive to such therapy. There is a pressing need for mechanism studies on this subject and potential mechanisms for synergistic effects. In the past 10 years, we and other laboratories have shown that anti-EGFR, anti-HER2, anti-CD20, and anti-CD47 in combination with immune checkpoint blockade could have synergistic effects in host adaptive anti-immune responses and tumor eradication. The mechanisms of such synergistic effect may be complicated. Antibodies targeting to tumor cells can not only reduce tumor burden but also change tumor microenvironment. Some antibodies, such as anti-Her2/neu, can increase IFN through MyD88, while others, such as anti-CD47, induce IFN through STING pathway. Our study also demonstrates that CTLA4 is a major immune suppressor in A20 tumors and leads to anti-CD20 resistance. Anti-CTLA-4 and anti-CD20 combined treatment overcomes adaptive resistance and prevents relapse in the mouse B-cell lymphoma model (Ren *et al.* 2017). But the mechanism is unclear. In another combination therapy, PD-L1 blockade could enhance the antitumor efficacy of anti-CD20–IFNα and reduce relapse rates for advanced large tumors that are resistant to either anti-CD20 Ab or the anti-CD20–IFNα fusion protein (Liao *et al.* 2017). IFN can increase PD-L1 expression on tumor cells and antigen presentation, each of which represents distinct mechanism of tumor control. PD-L1 on tumor cells may prevent T-cell-mediated killing, while PD-L1 on antigen-presenting cells may suppress T-cell reactivation. The role of PD-L1 on draining LN is unclear.

In our most recent study, Tang *et al.* demonstrated that anti-PD-L1 is significantly accumulated in tumors after systemic treatment and could be utilized to deliver immunomodulatory molecules (Tang *et al.* 2018), such as IFN–anti-PD-L1, specifically into tumor tissues (unpublished data from Yang-Xin Fu’s group). IFN–anti-PD-L1 may elicit a positive feedback loop to enhance targeting effects by upregulating PD-L1 expression, which is beneficial for treating tumors with lower levels of PD-L1. This strategy of PD-L1 antibody armed with IFN can overcome resistance to checkpoint blockade therapy in advanced tumors.

### Local radiotherapy can overcome PD-L1 resistance

Radiotherapy (RT) is a cancer treatment that employs high dose of radiation to kill tumor cells and reduce tumor burden. Not only tumor cells but also stromal cells at the tumor site can be affected by radiation. Radiation-damaged cancer cells release tumor antigens, DNA and RNA that are captured by antigen-presenting cells to promote the priming and activation of cytotoxic T cells, and to facilitate further infiltration of immune cells. Radiation-induced inflammatory response may result in multiple IR-resistant signaling that facilitate tumor relapse (Barcellos-Hoff *et al.* 2005), including enhancement of PD-L1/PD-1. Thus, application of radiation therapy with TTmAb and checkpoint blockade may result in synergistic effects for cancer regression. Animal studies and clinical trials have been taken to explore the most effective combinations, which may depend on the tumor type, specific immunotherapy, and optimal timing. In a study of radiation therapy combined with checkpoint blockades, anti-CTLA4 functions most effectively when administrated ahead of radiation, but anti-OX40 must be given after radiation to improve treatment efficacy in a mouse tumor model (Young *et al.* 2016). Another study showed that PDL1 blockade could overcome radiotherapy resistance only when anti-PDL1 was given concurrently with radiation, not a week before or after radiation (Dovedi *et al.* 2014).

Our previous study shows that upregulation of the PD-L1/PD-1 in tumor after IR inhibits radiation-induced anti-tumor immune responses and facilitates tumor relapse. Combination of IR with PD-L1 blockade results in the elimination of MDSCs by T-cell-derived TNF (Deng *et al.* 2014). This rational design of RT in combination with anti-PD-L1 should be referred to and applied in clinical cancer treatment. The optimization of radiation dose and timing is critical for enhancing the effectiveness of individual-based combination treatment.

### TTmAb combined with small molecule chemo- and targeted therapy

Antibody treatment in combination with multiple chemo-therapeutic agents has been investigated in a number of tumor models (Pegram *et al.* 1999, 2004). There is no conclusive result for whether chemotherapy synergizes with anti-HER2/neu antibody (Hudis 2007; Piccart-Gebhart *et al.* 2005; Romond *et al.* 2005). Our previous studies have shown that the timing of chemotherapy administration is critical for synergistic effects when combined with anti-Her2 and anti-CD47 therapy (Liu *et al.* 2015; Park *et al.* 2010). Pretreatment with chemotherapy can enhance the anti-tumor effect of anti-Her2 and anti-CD47 therapy, whereas chemotherapy applied following anti-Her2 or anti-CD47 treatment abolishes the tumor regression induced by either single treatment and even destroyed the tumor-specific T-cell memory responses (Liu *et al.* 2015; Park *et al.* 2010).

Molecular targeted therapy functions through blocking the growth of cancer cells by interfering with tumor-specific targeted molecules needed for oncogenesis and tumor growth. Tyrosine kinase inhibitors that target EGFR family members are among the most successful targeted cancer therapies (Arteaga and Engelman 2014; Scaltriti and Baselga 2006; Wieduwilt and Moasser 2008). Although the standard HyperTKI (low dose with a high frequency) regimen has shown a promising initial anti-tumor effect, tumor relapse happens to almost all patients eventually within about 1 year. Besides improving response rates, significant reducing tumor relapse rates have been a big challenge for EGFR TKIs treatment.

Immunotherapy, such as nivolumab or pembrolizumab, has been approved as the standard second-line or third-line treatment for TKI-resistant patients with high-level PD-L1 expression in tumors. Unfortunately, recent clinical trials were prematurely terminated due to significant side effects from checkpoint blockade combined with prolonged treatment of EGFR TKIs, which has achieved higher response rates (Ahn *et al.* 2017). Therefore, safety and efficacy must be carefully evaluated for development of the combination of EGFR TKI and immunotherapy.

## Summary and Perspectives

Recent work exploring the role of the tumor-targeting Ab in cancer therapy has yielded significant progress. Our laboratory’s research and the work of other research groups show that tumor-targeting Ab can be effectively combined with traditional small molecule chemo- and targeted therapy as well as newly developed checkpoint blockades, thus providing new potential for creating highly specific therapies. By targeting tumors, these new therapies would address the fundamental mechanism behind tumor growth and metastasis, namely breaking immune tolerance. It is the TTmAb that bridges the innate and adaptive immunity to modulate tumor microenvironment, overcomes drug resistance, and induces effective anti-tumor response to achieve tumor regression. This is the underlying principle behind the ongoing interest in combining targeting Ab with radiation and small molecule chemo- and targeted therapies.

Although this review mainly focuses on the functional studies and the clinical application of tumor-targeting antibodies, recent evidence has shown further development of such antibody fusion proteins armed with inflammation cytokines, including IFN, LIGHT, and IL-2. Moreover, immune checkpoints have been deemed critical for the cancer immune tolerance. As such, tumor-targeting checkpoint blockade would be a worthy direction for extensive future study. Exploring the synergistic treatment effect between targeting antibodies, immune cytokines, checkpoint blockade, radiotherapy, and small molecule chemo- and targeted therapies holds great promise for elucidating the underlying immunology mechanisms as well as developing effective therapies for a broad range of malignant diseases.
